# Comparative Hemolymph Proteomic and Enzymatic Analyses of Two Strains of* Rhipicephalus (Boophilus) microplus* Ticks Resistant and Susceptible to Ixodicides

**DOI:** 10.1155/2018/9451547

**Published:** 2018-06-11

**Authors:** H. Aguilar-Díaz, M. Esquivel-Velázquez, R. E. Quiroz-Castañeda, E. Miranda-Miranda, R. J. P. Conde-Baeye, M. Cobaxín-Cárdenas, P. Ostoa-Saloma, R. Cossío-Bayúgar

**Affiliations:** ^1^Unidad de Artropodología, Centro Nacional de Investigación Disciplinaria en Parasitología Veterinaria, Instituto Nacional de Investigaciones Forestales, Agrícolas y Pecuarias, INIFAP. Carr. Fed. Cuernavaca-Cuautla No. 8534, Jiutepec, Morelos, 62550, Mexico; ^2^Dirección de Investigación, Hospital General de México “Dr. Eduardo Liceaga”, Dr. Balmis 148, Col. Doctores, Ciudad de México, 06720, Mexico; ^3^Unidad de Anaplasmosis, Centro Nacional de Investigación Disciplinaria en Parasitología Veterinaria, Instituto Nacional de Investigaciones Forestales, Agrícolas y Pecuarias, INIFAP. Carr. Fed. Cuernavaca-Cuautla No. 8534, Jiutepec, Morelos, 62550, Mexico; ^4^Centro de Investigaciones Sobre Enfermedades Infecciosas, Instituto Nacional de Salud Pública, Av. Universidad 655, CP 62501 Cuernavaca, Morelos, Mexico; ^5^Instituto de Investigaciones Biomédicas, Universidad Nacional Autónoma de México, Ciudad Universitaria, México 044510, D.F., Mexico

## Abstract

The cattle tick* Rhipicephalus (Boophilus) microplus* is one of the most harmful ectoparasites affecting bovines worldwide. It represents a major threat to livestock industry due to the economic losses caused and diseases associated with these ticks. The most important tick control strategy has been the use of ixodicides, resulting in chemically resistant tick populations. It is necessary to understand the mechanisms that result in resistance so as to create new strategies increasing the lifespan of ixodicides or finding alternative targets to produce new acaricides. In this paper, in order to obtain an insight into the mechanisms that govern ixodicides resistance, we will compare the hemolymph proteome of two tick* R. microplus* strains, one susceptible (MJ) and one resistant (SA) to ixodicides, using HPLC and 2D electrophoresis. Significant differences were found in protein content between strains using HPLC. 2D electrophoresis revealed that 68 hemolymph protein spots were common between strains; however, 26 spots were unique to the susceptible strain MJ and 5 to the resistant strain SA. The most distinctive protein spots on the preparative gels were selected for further analyses. Nine protein spots were identified by mass fingerprinting, * *revealing proteins that may have a role in the ixodicides resistance or susceptibility. In this paper, we present the tick hemolymph proteome revealing a set of proteins which suggest a possible role in tick detoxification.

## 1. Introduction

Ticks are considered obligate haematophagous ectoparasites that infest wild and domestic animals. They are responsible for significant economic losses mainly associated with mortality and morbidity of livestock animals [1].

In cattle, the worldwide distributed tick* Rhipicephalus (Boophilus) microplus* (Acari: Ixodidae) represents an important threat to animal health and production [2]. This tick is especially distributed in tropical and subtropical environments where it infests bovines and is considered one of the most harmful vectors of tick-borne diseases in cattle [3]. In endemic areas with* R. microplus*, major economic losses are due to anemia which leads to a reduction in weight gain and milk production. Indirectly, the main risk is the transmission of pathogens such as* Anaplasma *and* Babesia *spp*., *among other bacterial and fungal infections [4].

Tick infestations in bovine cattle are usually treated with chemical ixodicides applied directly to the skin of animals. However, uncontrolled and excessive use of these compounds exerts a selection pressure on those ticks with ixodicides multiresistance. This is a well-known widespread phenomenon that increases the occurrence of food and environmental contamination [2, 5–7]. In general, the mechanism of resistance is usually associated with increased metabolic detoxification or target site modification, as previously described [7–10]. Nonetheless, there is still much more to elucidate regarding tick physiology and immunology. Therefore, efforts can be directed to identify novel biomolecules candidates for tick control using other approaches, such as genomics, transcriptomics, and proteomics (omics). The main contribution of genomic approaches in the study of vector-borne diseases is the design of new peptides identified by* in silico* analysis of transcriptomic and genomic data so as to develop novel vaccines against ticks [11, 12]. For the last ten years, “omics” approaches have provided valuable information regarding tick–host interface immunobiology, mainly by means of genomic, transcriptomic, and proteomic data of several species of ticks, regularly using larvae or engorged females [3, 13–16]. However, until the last year, the first tick* I. scapularis *genome was released and new possibilities of study were opened, especially those related to host–tick–pathogen interactions, parasitic processes unique to ticks, tick reproduction, and so forth [17, 18].

In* R. microplus, a *proteomic analysis of saliva (sialomes) showed differences in saliva protein profiles and composition between partially engorged (PE) and fully engorged female ticks. This was the first proteomic study of tick saliva that contributed to understanding the role of tick salivary modulators in immunological defense of ticks. Proteins identified in this proteomic analysis included microplusin-like proteins, lipocalins, serpins, and hemelipoprotein (HeLp) [3, 7]. Hemelipoproteins have been reported as the most abundant proteins in tick saliva and in hemolymph in several tick species, in which transcriptional profile and protein localization analyses suggested that this protein may play vital roles in tick feeding and survival [19, 20]. Other proteomic studies have also been performed in* Amblyomma americanum *(salivary secretions)*, I. scapularis *(saliva protein composition),* Ornithodoros moubata *(tick saliva), and* R. sanguineus *(sialoma) [19, 21–23]. So far, our knowledge of hemolymph proteins in ticks is still scarce, and its relevance lies in the roles this fluid plays in this arthropod. Hemolymph is a fluid that bathes all tissues in ticks. It is the first source of nutrients, transports molecules and hormones, and provides protection to pathogen agents to which ticks are exposed [24]. The hemolymph, as a vehicle of transport of factors related to humoral immune response, has been explored in insects and crustaceans but not in ticks, where it may have a significant role in defense and protection not only to microbial invasions but also to environmental factors such as pesticides [25]. Likewise, the relationship of the hemolymph, immune response, and susceptibility-resistance genotype still remains unclear. Also, to date, no “omics” studies on tick hemolymph have been reported, and much less, the role of hemolymph is unknown. In this work, we present proteomic profiles of hemolymph from resistant and susceptible ticks. The analysis of the proteomic profiles provides useful information for understanding the relationship between ixodicides and the resistance/susceptibility mechanisms of ticks along with the identification of proteins which may have key roles. The goal of this study is to find those proteomic differences between the hemolymph from ticks that are ixodicides-resistant when compared to reference strains susceptible to SDS/PAGE.

## 2. Materials and Methods

### 2.1. Tick Strains

The ixodicide susceptible* R. microplus* tick strain Media Joya (MJ) and the multiple ixodicides-resistant tick strain San Alfonso (SA) [26] were used in this study (Table 1). The strains have been maintained in controlled infested bovines for many generations and used as reference for the tick ixodicides resistance monitoring programs of the Mexican Federal Government. The resistant strain SA was reared and maintained at Departamento de Ectoparásitos y Dípteros del Servicio Nacional de Sanidad, Inocuidad y Calidad Agroalimentaria (SENASICA-SAGARPA), and susceptible strain MJ at Centro Nacional de Investigación Disciplinaria en Parasitología Veterinaria (CENID-PAVET, INIFAP). Each reference strain was obtained by infesting a bovine with 2 × 10^4^ larvae, aged between 10 and 15 days, and then engorged tick females were collected 21 days after infestation and placed in Petri dishes in groups of 10 for immediate hemolymph extraction as reported previously by [27].

### 2.2. Bioassays on Ixodicides Discriminant Doses

Bioassays were run as reported previously by [28]. The ixodicides were diluted in trichloroethylene (Sigma-Aldrich) at the following concentrations: coumaphos 0.2%, chlorfenvinphos 0.2%, diazinon 0.08%, chlorpyrifos 0.2%, cypermethrin 0.05%, deltamethrin 0.09%, and flumethrin 0.01%. One milliliter of each dilution was applied evenly to a 7 × 9 cm piece of filter paper (Whatman, Sigma-Aldrich). The trichloroethylene was allowed to evaporate from the filter paper for 2 h. The filter papers were then folded in half and sealed on the slides with clips, which formed a packet into which approximately 100 larvae were placed and then the top of the packet was sealed with another clip. The packets were kept at 27°C and 92% relative humidity for 24 h. The packets were removed from incubation and opened, live and dead larvae were counted, and the data was processed as percentage of mortality for each tick group under every ixodicide concentration.

### 2.3. Protein and Hemolymph Extraction and Quantification

Engorged tick females were placed in Petri dishes and observed under a dissecting microscope. The hemolymph was obtained by performing an incision with sterile scalpels in the anterior segment of ticks, very carefully to not damage any internal organ. The hemolymph was mixed with an equal volume of collection buffer (98 mM NaOH, 186 mM NaCI, 1.7 mM EDTA, and 41 mM citric acid, pH 4.5), in sterile tubes (Stoepler et al. 2012) and protease inhibitor (Roche). Then, the hemolymph was frozen and thawed three times by placing the tubes in liquid nitrogen for 30 seconds, followed by incubation at 37°C for 3 minutes, in order to lyse any cell. Extracts were then centrifuged at 14000xg for 10 minutes at 4°C. The supernatant was collected, quantified by conventional Bradford method (Bio-Rad), and stored at -70°C [29]. This protocol allowed the collection of 10-20 *μ*l of hemolymph per tick, reducing free of detritus cell, melanization rest, tissue debris, or other contaminants.

### 2.4. HPLC Sample Preparation

The hemolymph from resistant strain SA and susceptible strain MJ was collected as previously described and supplemented with protease inhibitors with 0.01% phenyl thiourea, 1 *µ*g/ml leupeptin 10 *µ*l/ml, TLCK (L-1-chloro-3-(4-tosylamide)), and 10 *µ*l/ml and 1 *µ*l/ml PMSF (all supplied from Roche). The resultant supernatants were centrifuged at 12000xg for 30 minutes and then adjusted to 0.1% with trifluoroacetic acid (TFA) (Sigma-Aldrich).

### 2.5. HPLC Fractioning

The samples were applied on a C-18 reverse phase HPLC column on an Agilent 1100 series HPLC apparatus and eluted with a 0 to 50 % acetonitrile gradient (ACN) (SIGMA) with 25 minutes of collection and then with 50 to 100% ACN gradient within 20 minutes of collection. The collected fractions were lyophilized and resuspended in 50 *μ*l of phosphate-buffered saline 1X.

### 2.6. Zymograms Assays

70 ng of protein extract from each hemolymph sample was electrophoresed as previously described [30]. After electrophoresis, gels were stained for detection of catalase and phosphatase enzymatic activity as described previously [31]. 70 ng of each protein extract was assayed by the Sigma® catalase assay kit and Sigma® phosphatase colorimetric assay kit according to the manufacturer's instructions. Total proteins were stained by Coomassie brilliant blue R250 (Sigma-Aldrich) 0.5% for reference.

### 2.7. Determination of Phenol Oxidase (PO) Production

**Figure 1:**
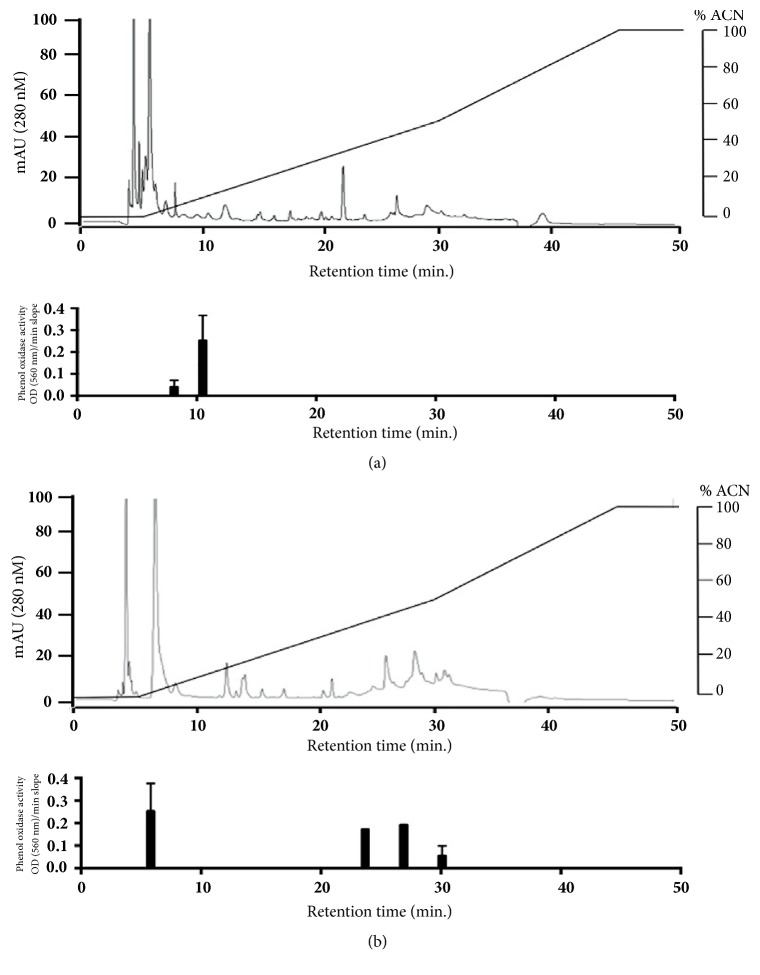
HPLC chromatography profiles of susceptible MJ (a) and resistant SA tick strains (b). At higher concentrations of ACN (acetonitrile), multiple peaks eluted in susceptible strain MJ ((a), upper), while at low concentrations of ACN, peaks were observed in resistant strain SA ((b), upper). The oxidizing activity exhibited through L-DOPA oxidation is depicted below the chromatograms ((a) and (b), lower); the graph exhibits an increase of oxidative activity approximately at 10 min retention time in the susceptible MJ when compared to the resistant SA; additionally, the resistant strain SA exhibits several peaks of oxidative activity which are not present in the susceptible strain MJ during the 20 to 30 min retention time.

Hemolymph fractions from HPLC fractioning were probed for PO activity using l-DOPA (dihydroxyphenylalanine, Sigma-Aldrich) chromogenic reaction, as described elsewhere (29). 5 *µ*l of the HPLC fractions was mixed with 50 *µ*l of 0.13 mg 3-4-dihydroxy-L-phenylalanine/ml distilled water. The solution was incubated for one hour at ambient temperature and the 620 nm absorption was recorded every ten minutes. The slope of the 620 nm OD increase of every fraction tested was compared with the control reaction (H_2_O) and samples with consistent OD increases were graphed in Figure 1.

### 2.8. Two-Dimensional Electrophoresis (2D-E) and Gel Analysis

For analytical 2D gels, 35 *µ*g of hemolymph proteins was loaded onto broad range lineal 7 cm IPG strips (pH 3-10, GE Healthcare) in rehydration buffer (8 M urea, 2% w/v CHAPS, 0.8% w/V DTT, 1.6% v/v Bio-Lyte Broad Range Ampholytes, and 0.002% w/v bromophenol blue) and left to rehydrate for 12 hours at room temperature. Isoelectric focusing was performed with PROTEAN i12 IEF System for a total of 10,000 V-h in four steps: (1) 125 V for 2 h, rapid; (2) 250 V for 20 min, gradual; (3) 4000 V for 2 h, rapid; and (4) 4000 V for 10,000 V-h, rapid.

Strips were then equilibrated with 1% w/v DTT (Promega) and then with 2.5% w/v iodoacetamide (Sigma-Aldrich) in fresh equilibration buffer (6 M urea, 0.375 M Tris-HCl, pH 8.8 (Sigma-Aldrich), 2% w/v SDS, and 20% v/v glycerol) for 10 minutes each under constant rocking. The second-dimension electrophoresis was performed in 4-20% precast gels (Bio-Rad) in denaturing conditions. Gels were then fixed with buffer containing acetic acid 7% and methanol 10% for 30 minutes.

Protein spots were stained with Sypro Ruby (Bio-Rad) according to manufacturer's instructions. Stained gels were scanned with Typhoon FLA 9500 imaging system (GE Healthcare) and spot analysis was performed with PDQuest software (Bio-Rad). All samples were run in triplicate. Only spots that replicated in at least two out of the three replicates were considered for analysis. Resistance and susceptibility conditions were compared and spot differences were identified.

In order to pick spots for protein identification, samples were run in duplicate. 144 *µ*g of hemolymph proteins from resistant strain (SA) ticks and 64.88 *µ*g from susceptible (MJ) ticks were loaded onto IPG strips (pH 3-10, lineal) and proteins were separated as previously stated. Gels were fixed and stained with Coomassie G-250. Gel spot picking was performed manually with custom-cut pipet tips over a light box.

### 2.9. Protein Identification Analysis

A total of 13 protein spots were selected for digestion sequence and mass spectrometry analysis at the University Proteomics Laboratory at Biotechnology Institute of the National Autonomous University of México. In gel digestion of proteins was performed with trypsin (Promega) in reaction buffer containing ammonium bicarbonate 50 mM for 18 hours at 37°C. Peptides were desalted with Zip Tip C18 (Millipore) and applied to a LC-MS system (Nanoflux Pump EASY nLC II and LTQ-Orbitrap Velos, Thermo Fischer) with nanoelectrospray ionization (ESI). Proteins were identified automatically by Proteome Discoverer 1.4 software through the search engines SEQUEST-HT.* Boophilus, Rhipicephalus,* and protein databases obtained from NCBI were used for * *identity searches. Spot analyses were performed with InterPro, SMART (Simple Modular Architecture Research Tool), ProtoNet, Pfam, and SPRINT software.

## 3. Results

### 3.1. Bioassays on Ixodicide Discriminant Doses

Resistant strain SA was assayed by using the larval package procedure for discriminant ixodicides doses previously reported [28]; the bioassay results were used as selection criterion for ticks from where the hemolymph was obtained. Once the packets with ixodicides were opened, live and dead larvae were counted and percentages of mortality were calculated. In Table 1, percentages of mortality are shown for susceptible strain MJ and resistant strain SA. 100% mortality of susceptible strain MJ was observed with all ixodicides assayed, including organophosphorous, pyrethroids, and amidines. Multiple ixodicides-resistant strain SA showed resistance to the three pyrethroids used (cypermethrin, flumethrin, and deltamethrin) with 0% mortality; on the contrary, 100% mortality was observed with two organophosphorous compounds (diazinon and chlorpyrifos) and 73.24% with coumaphos. This strain also showed 10.76% mortality with amidines (diazinon).

### 3.2. HPLC Chromatography

Spectroscopy profiles of hemolymph at 280 nm showed different patterns (Figure 1); the profile of the susceptible strain MJ exhibited an increasing complexity when eluted at higher ACN percentage (Figure 1(a), upper), whereas the profile of the resistant strain SA exhibited a more complex pattern when eluted at lower ACN percentage (Figure 1(b), upper).

### 3.3. Zymograms Assays

Catalase and phosphates are key enzymes during xenobiotics metabolism of most metazoan organisms; these enzymes, in combination with other xenobiotic metabolizing enzymes, are required by pesticide resistant arthropods for metabolic degradation of toxic chemicals into less damaging compounds. We considered a possible differential expression of these enzymes when comparing susceptible against resistant ticks.

#### 3.3.1. Phosphatase Activity

**Figure 2:**
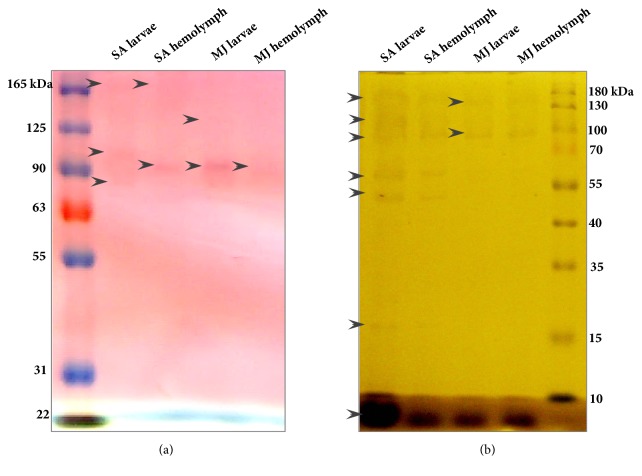
Phosphatase and catalase activity gels of hemolymph proteins of susceptible MJ and resistant SA tick strains. (a) In phosphatase zymogram, two protein bands in hemolymph of resistant strain SA were observed while only one protein band was observed in susceptible strain MJ. (b) In catalase zymogram, the protein band patterns of resistant strain SA and susceptible strain MJ hemolymph are different. In resistant strain SA, seven protein bands with catalase activity were observed and only three of these bands were observed in susceptible strain MJ. Arrows indicate protein band position. Larvae hemolymphs of resistant SA and susceptible MJ strains were used as a known positive control of these activities.

The protein bands pattern with phosphatase activity varied between hemolymphs from susceptible MJ and resistant SA strains as observed in Figure 2(a).

In hemolymph extracts from resistant strain SA, protein bands with enzymatic activity were observed (molecular weight approx. 90 and 170 kDa) and only one protein band with phosphatase activity was observed in susceptible strain MJ hemolymph (molecular weight approx. 90 kDa). A different protein band pattern was also observed between resistant strain SA and susceptible strain MJ larvae extracts.

#### 3.3.2. Catalase Activity

Catalase activity was assessed in a zymogram of hemolymph and larvae extracts of both resistant SA and susceptible MJ strains as shown in Figure 2(b).

In hemolymph extracts of resistant strain SA, catalase activity in hemolymph was assigned to seven protein bands with enzymatic activity (molecular weight approx. 8, 17, 50, 60, 90, 110, and 170 kDa), while in susceptible strain MJ hemolymph extracts, this number of bands decreased to three (molecular weight approximately 8, 90, and 170 kDa).

### 3.4. Phenol Oxidase (PO) Activity

The HPLC fractions showed differential PO activity: susceptible strain MJ hemolymph showed two fractions showing activity eluting from the column around 10% ACN (Figure 1(a), lower). Resistant strain SA hemolymph fractionation eluted samples with oxidizing activity in the first fractions (0.5% ACN elution) but also at 33%, 40%, and 50% ACN (Figure 1(b), lower). These multiple peaks of oxidizing activity in the resistant strain SA hemolymph might reflect distinct proteolytic activation of the same enzyme (the hydrophilic protein peak with oxidizing activity eluted at 0.5% ACN elution for the resistant strain SA and 10% ACN for the susceptible strain MJ). Also, the more hydrophobic peaks showing oxidizing activity could correspond to a set of enzymes unique to the susceptible strain MJ.

### 3.5. Protein Identification Analysis

**Figure 3:**
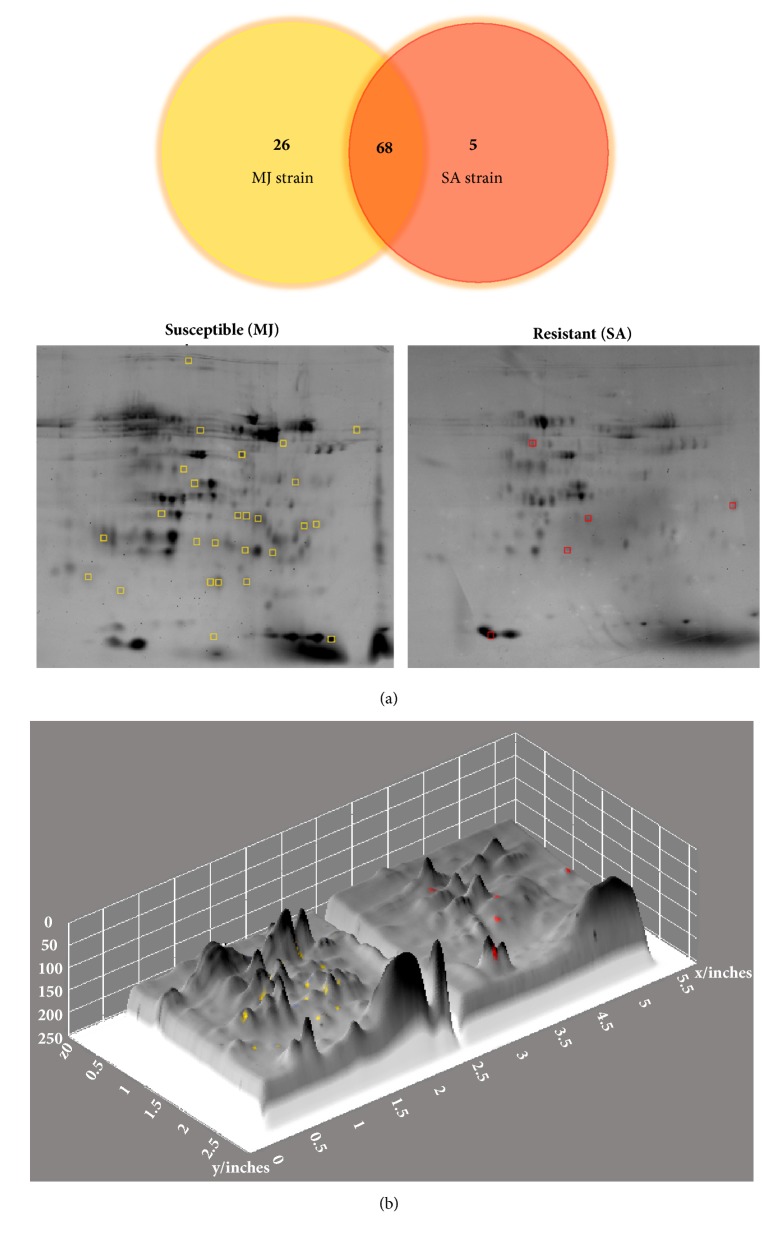
2D electrophoresis gel of hemolymph proteins of susceptible MJ and resistant SA tick strains. (a) 26 proteins were identified by PDQuest software in susceptible strain MJ and 5 proteins in resistant strain SA. 28 proteins were identified as common proteins of both strains. Yellow and red squares correspond to those protein spots that were selected for sequencing. (b) 3D surface plot of 2D electrophoresis. Protein concentration differences are observed in each plot.

**Figure 4:**
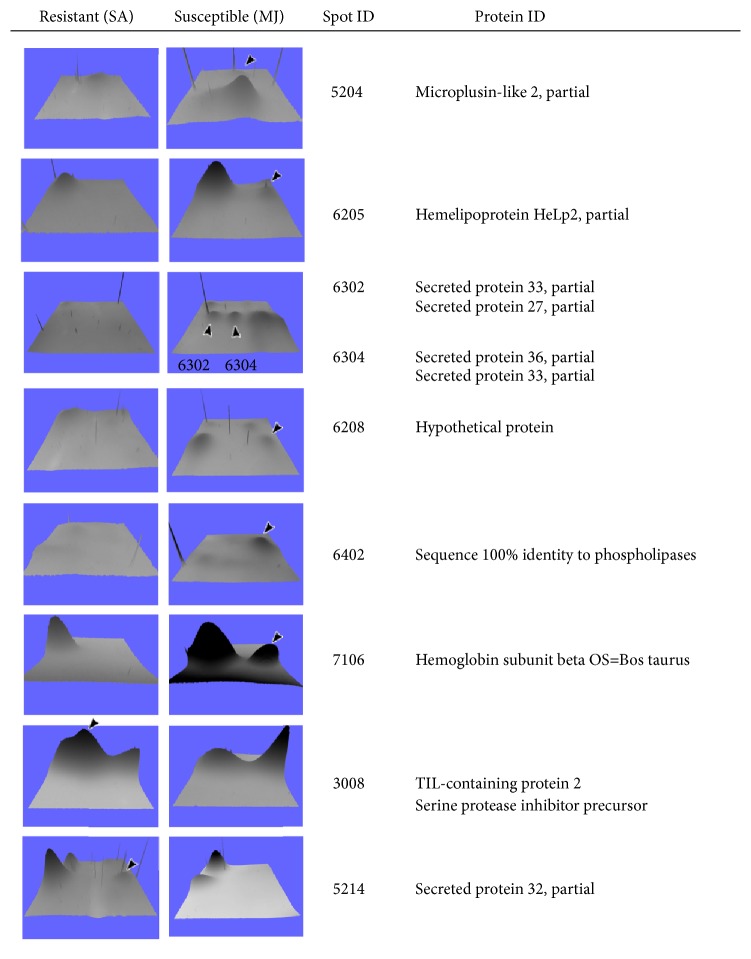
3D visualization of the identified spots. The same area from representative gels from resistant SA and susceptible MJ tick strains was selected and visualized as 3D graphs. The identified spots are indicated with arrows in the images and protein identity is indicated to the right.

In order to further explore the differences in the protein from resistant strain SA and strain susceptible MJ* R. microplus*, we compared their proteomes by means of 2D electrophoresis. The proteome images were analyzed with PDQuest software to detect spots and compare the triplicates. 68 protein spots were common to both strains, 26 spots were unique of the susceptible strain MJ, and 5 were unique to the resistant strain SA. From the spots unique to each strain, we selected those clearly visible in preparative gels. The selected spots were gel-picked and sent for identification by mass fingerprinting (Figure 3). A total of 13 spots were sent for identification, from which 9 were successfully identified (Figure 4, Table 2).

## 4. Discussion

The massive use of ixodicides from the control of the cattle tick is collaterally producing the right conditions for the selection of resistance. This adaptation is attributed to resistance mechanisms that ticks have to use in order to survive in new and hostile environments, which results in serious problems of chemical tick control for the cattle farmers [46]. Hemolymph is required by all arthropods as blood is for vertebrate organisms; it provides osmotic balance and a mechanism of nutrients and oxygen distribution and also acts as the liquid medium for the propagation of circulating cells and molecules including those related to the immune system and detoxification of pesticides. In this work, we explored the role of hemolymph proteins of susceptible and resistant tick strains with a proteomic approach.

Besides the immunogenic function attributed to hemolymph during pathogen invasion, it also possesses elements used to detoxify ticks when they are exposed to ixodicides. This mechanism is a complex process that includes enzymatic reactions mediated by phosphatases, catalases, and glutathione S transferases [47, 48], which modify metabolic pathways resulting in metabolic resistance. During this study, we used SDS-PAGE zymograms and found that those polypeptide bands that exhibit specific catalase activity were different when comparing zymograms from resistant strain SA and susceptible strain MJ. The strain SA is regarded as pyrethroid resistance reference strain. According to previous studies, this type of resistance is based on overexpression of oxidases and cytochrome P450 monooxygenases acting in a functional enzymatic combination [49, 50]. The catalase and phosphatase zymogram analysis showed that both strains have a differential pattern of protein bands with enzymatic activity that may be related to ixodicides resistance; whether this is an effective biochemical marker for detection in cattle tick is to be further assessed in future studies, besides a significant role of other enzymes such as glutathione S transferases, oxidases, esterases, and cytochrome P450 monooxygenases [50–52] in ixodicides resistance of cattle ticks. In fact, other oxidase enzymes such as phenol oxidase (PO) have been reported in insects (*Anopheles *spp.*, Triatoma *spp.) as part of the melanization process of the protective immune response. However, the role of PO in ticks still remains unknown [53]. So, we tried to correlate the enzymatic activity involved in detoxification with resistant and susceptible phenotypes. We observed higher oxidative enzymatic activity in the hemolymph of the susceptible strain MJ, when compared to the resistant strain SA. It is plausible that cypermethrin/flumethrin/deltamethrin resistance presented in the susceptible strain SA reduced PO activity may be associated with the necessity to metabolically modify pesticides in order to acquire an increased toxic effect [50]. Also, HPLC chromatography profiles of susceptible strain MJ and resistant strain SA revealed that a higher number of peaks were eluted at higher ACN concentrations in susceptible strain MJ; on the contrary, a more complex HPLC profile was obtained at low ACN concentration in resistant strain SA. These results suggested that hemolymph of resistant strain SA has more hydrophilic components than hemolymph of susceptible strain MJ.

In order to explore hemolymph protein differential expression, we carried out 2D electrophoresis. Different spots were identified in protein maps of both strains. We observed a remarkable difference in the amount of spots, especially in resistant strain SA where this number was significantly lower than in the susceptible strain MJ. So far, evolution theories propose that organisms exposed to adverse environmental conditions have to deal with a trade-off of energy and having limited resources for growth, reproduction, and defense to cope with those conditions [54]. Although this condition may be advantageous under certain circumstances, it also can simultaneously reduce several functions related to physical and chemical limitations, as well as to genetic mechanisms and energy assignment (antagonistic pleiotropy) [55, 56]. It is likely that growth, reproduction, and defense performance are linked to a reduced capacity in ixodicides resistance. According to this, the diversity in the number of spots in protein maps (resistant and susceptible phenotypes) suggests that such high number observed is not necessarily associated with a robust resistance mechanism, since we found that the resistant strain SA has fewer spots than the susceptible strain MJ. At this point, we only identified the protein maps of ticks under two conditions; however, the amount of proteins that may directly be associated with resistance or susceptibility is not clear.

Proteomic comparison analysis of both strains revealed that 68 proteins are common to resistant SA and susceptible MJ strains, 26 spots are only present in susceptible strain MJ, and 5 spots are present in resistant strain SA. Of those spots that were exclusively present in susceptible strain MJ, we identified a protein with 100% identity to phospholipases that has not been previously reported. Another protein identified was hemelipoprotein HeLp2 that was overexpressed in susceptible strain MJ. This protein has been reported as part of the tick sialome and considered one of the more abundant proteins in salivary glands of ticks and also of the hemolymph [3]. Since expression of HeLp2 is lower in resistant strain SA, it is likely that this protein does not have a crucial role in resistance mediated by hemolymph components.

In the susceptible strain MJ, we also identified microplusin-like 2 protein, one of the main components of innate immune response [45]. We speculate that this protein is present under susceptibility conditions and not in resistance conditions, probably as a result of the trade-off that ticks would be performing when they are exposed to ixodicides. Finally, protein SUSMJ-6304 identified in the susceptible strain MJ is also relevant, since* in silico* analysis showed that sequence matched with Cu-Zn superoxide dismutase (SOD) in an arthropods database (UnitProt), an enzyme involved in cellular detoxification processes by removing free radicals of many species. Nevertheless, it is known that SOD is highly present in sialome of ticks, so probably the enzyme identified in the susceptible MJ and resistance SA strains would be a leakage from salivary glands to hemolymph. Previously, we measured SOD activity by enzymatic assays of hemolymph of both strains with negative results (data not shown). However, many reports have shown the role of free radicals in the inhibition of some insecticides, which may explain why the spot SUSMJ-6304 of susceptible strain MJ has a relative abundance higher than the resistance strain SA.

In this work, we report the first proteomic approach of tick hemolymph in Mexican* R. microplus *strains. We identified both previously and never reported proteins, as well as enzymatic activities related to ixodicides susceptibility or resistance, which may be used as future targets for diagnostic, drugs, and vaccines design to contribute to ticks control.

Future perspectives of this work will be driven to develop targets that could be used in prevention and therapeutic methods for a tick integral control.

## Figures and Tables

**Table 1:** Percentage of mortality of susceptible MJ and resistant SA tick strains to ixodicides.

**Percentage of mortality (%)**							
Tick Strain	Organophosphorous			Pyrethroids			Amidines
	Coumaphos	Diazinon	Chlorpyrifos	Cypermethrin	Deltamethrin	Flumethrin	Amitraz
Susceptible (MJ)	100	100	100	100	100	100	100

Resistant (SA)	73.24	100	100	0	0	0	10.76

**Table 2:** Identified proteins in hemolymph from susceptible MJ and resistant SA *R. microplus* tick strains.

**Spot ID**	**Accession number **	**Description**	**Score (%)**	**Coverage (%)**	**Theoretical MW (kDa)/IP**	**Function**	**Reference**
SUSMJ-5204	JAC59075.1 (GenBank)	Microplusin-like 2, partial (*Rhipicephalus microplus*)	495.58	31.63	10.7/6.98	Antimicrobial peptide isolated from the cattle tick *R. microplus*. Its copper-chelating ability is putatively responsible for its bacteriostatic effect.	[32]

SUSMJ-6205	JAC59122.1 (GenBank)	Hemelipoprotein HeLp2, partial (*Rhipicephalus microplus*)	29.67	5.99	30.5/6.65	Protein involved in heme transports from hemolymph to tissues, suggesting to be an essential adaptation to the loss of the heme synthesis pathway	[33]

SUSMJ-6208	JAP78009.1 (GenBank)	Hypothetical protein (*Rhipicephalus appendiculatus*)	7.25	20.12	18.9/6.77	Protein binding to EGF. The functional significance of EGF domains in what appears to be unrelated proteins is not yet clear. However, a common feature is that these repeats are found in the extracellular domain of membrane-bound proteins or in proteins known to be secreted	[34]

SUSMJ-6302	JAC59082.1 (GenBank) JAC59119.1 (GenBank)	Secreted protein 33, partial (*Rhipicephalus microplus*) Secreted protein 27, partial (*Rhipicephalus microplus*)	10.08 8.31	3.83 3.86	37.8/7.99 37.4/7.28	Leucine-rich repeat domain, L domain-like. Leucine-rich repeats (LRR) consist of 2-45 motifs of 20-30 amino acids in length that generally fold into an arc or horseshoe shape. LRRs occur in proteins ranging from viruses to eukaryotes, and appear to provide a structural framework for the formation of protein-protein interactions. Proteins containing LRRs include tyrosine kinase receptors, cell-adhesion molecules, virulence factors, and extracellular matrix-binding glycoproteins, and are involved in a variety of biological processes, including signal transduction, cell adhesion, DNA repair, recombination, transcription, RNA processing, disease resistance, apoptosis, and immune response	[35–38]

SUSMJ-6304	JAC59079.1 (GenBank)	Secreted protein 36, partial (*Rhipicephalus microplus*)	23.30	16.26	21.6/7.12	Copper/zinc superoxide dismutase (SOD). superoxide dismutases catalyse the conversion of superoxide radicals to molecular oxygen.	[32]
	JAC59082.1 (GenBank)	Secreted protein 33, partial (*Rhipicephalus microplus*)	16.45	3.83	37.8/7.99	Uncharacterized. The component of a membrane consisting of the gene products and protein complexes having at least some part of their peptide sequence embedded in the hydrophobic region of the membrane. Reported by Tirloni 2014.	[3]
	*De novo*	100% identity to phospholipases		100	---	Phospholipase-mediated phospholipid hydrolysis leads to the production of lipid mediators or second messengers that affect signal transduction, thus regulating a variety of physiologic and pathophysiologic processes.	[39]

SMJ-7106	P02070 (UniProtKB/Swiss-Prot) 2QSP (PDB) 2EU1 (PDB)	Hemoglobin subunit beta OS=Bos Taurus GN=HBB PE=1 SV=1 (HBB_BOVIN) C Chain C, Bovine hemoglobin At Ph 5.7 D Chain D, Crystal structure determination of goat hemoglobin (Capra hircus) at 3 Angstrom resolution	1919.94 163.98 1288.43	82.76 78.72 55.17	15.9/7.59 15.0/8.44 16.0/7.30	Haemoglobins (Hb) function is transport oxygen in blood plasma, and binds oxygen in the reduced [Fe(II)] state. (Hb) tetramer of two alpha and two beta chains, although embryonic and foetal forms can substitute the alpha or beta chain for ones with higher oxygen affinity, such as gamma, delta, epsilon or zeta chains. Hb transports oxygen from lungs to other tissues in vertebrates. Hb proteins are also present in unicellular organisms where they act as enzymes or sensors.	[40–42]

RESSA-3008	JAC59001.1 (GenBank)	TIL domain-containing protein 2 (*Rhipicephalus microplus*)	1193.73	52.94	9.2/5.77	Different analyses carried out show that thia protein contains a domain 25 -81 TIL (trypsin inhibitory-like) This domain is found in proteinase inhibitors, as well as in many extracellular proteins. The domain typically contains ten cysteine residues that form five disulphide bonds. The cysteine residues that form the disulphide bonds are 1-7, 2-6, 3-5, 4-10 and 8-9.	[3]
	ACV83329.1 (GenBank)	Serine proteinase inhibitor precursor (*Rhipicephalus microplus*)	872.04	67.06	9.3/6.24	Serpin family members have activity as inhibitors of serine proteinases, and serve in regulation of proteinase-mediated, such as inflammatory, and the immune response with consequent pathology in deficiency state.	[43]
	ABH10604.1 (GenBank)	Neutrophil elastase inhibitor (*Rhipicephalus microplus*)	210.52	21.15	11.5/6.51	In ticks, these inhibitors (due to their properties) play a role in the feeding process, however the crucial role is still unclear.	[44]

RESSA-5214	JAC59087.1 (GenBank)	Secreted protein 31, partial (*Rhipicephalus microplus*)	17.73	2	15.6/6.52	Unknown. Reported by Tirloni 2014.	[45]

## References

[1] Domingos A., Antunes S., Borges L., Rosario V. E. (2013). Approaches towards tick and tick-borne diseases control. *Revista da Sociedade Brasileira de Medicina Tropical*.

[2] Popara M., Villar M., Mateos-Hernández L., Fernández de Mera I. G., de la Fuente J. (2013). Proteomics approach to the study of cattle tick adaptation to white tailed deer. *BioMed Research International*.

[3] Tirloni L., Reck J., Terra R. M. S. (2014). Proteomic analysis of cattle tick Rhipicephalus (Boophilus) microplus saliva: A comparison between partially and fully engorged females. *PLoS ONE*.

[4] Jonsson N. N., Bock R. E., Jorgensen W. K. (2008). Productivity and health effects of anaplasmosis and babesiosis on Bos indicus cattle and their crosses, and the effects of differing intensity of tick control in Australia. *Veterinary Parasitology*.

[5] De La Fuente J., Rodríguez M., Redondo M. (1998). Field studies and cost-effectiveness analysis of vaccination with Gavac(TM) against the cattle tick Boophilus microplus. *Vaccine*.

[6] Lovis L., Reggi J., Berggoetz M., Betschart B., Sager H. (2013). Determination of acaricide resistance in rhipicephalus (Boophilus) microplus (Acari: Ixodidae) field populations of Argentina, South Africa, and Australia with the larval tarsal test. *Journal of Medical Entomology*.

[7] Parizi L. F., Pohl P. C., Masuda A. (2009). New approaches toward anti-Rhipicephalus (Boophilus) microplus tick vaccine. *Revista Brasileira de Parasitologia Veterinária*.

[8] Rosario-Cruz R., Almazan C., Miller R. J., Dominguez-Garcia D. I., Hernandez-Ortiz R., De La Fuente J. (2009). Genetic basis and impact of tick acaricide resistance. *Frontiers in Bioscience*.

[9] Guerrero F. D., Lovis L., Martins J. R. (2012). Acaricide resistance mechanisms in Rhipicephalus (Boophilus) microplus. *Revista Brasileira de Parasitologia Veterinária*.

[10] Li A. Y., Davey R. B., Miller R. J., George J. E. (2004). Detection and characterization of amitraz resistance in the Southern Cattle tick, Boophilus microplus (Acari: Ixodidae). *Journal of Medical Entomology*.

[11] Miller R., Estrada-Peña A., Almazán C. (2012). Exploring the use of an anti-tick vaccine as a tool for the integrated eradication of the cattle fever tick, Rhipicephalus (Boophilus) annulatus. *Vaccine*.

[12] Temeyer K. B., Chen A. C., Davey R. B., Guerrero F. D., Howell J. M., Kammlah D. M. (2013). Nuevos enfoques para el control de Rhipicephalus (Boophilus) microplus. *Revista Mexicana de Ciencias Pecuarias*.

[13] Ribeiro J. M. C., Labruna M. B., Mans B. J. (2012). The sialotranscriptome of Antricola delacruzi female ticks is compatible with non-hematophagous behavior and an alternative source of food. *Insect Biochemistry and Molecular Biology*.

[14] Cardoso F. F., Gomes C. C. G., Sollero B. P. (2015). Genomic prediction for tick resistance in braford and hereford cattle. *Journal of Animal Science*.

[15] Pagel Van Zee J., Geraci N. S., Guerrero F. D. (2007). Tick genomics: The Ixodes genome project and beyond. *International Journal for Parasitology*.

[16] Rodriguez-Valle M., Lew-Tabor A., Gondro C. (2010). Comparative microarray analysis of Rhipicephalus (Boophilus) microplus expression profiles of larvae pre-attachment and feeding adult female stages on Bos indicus and Bos taurus cattle. *BMC Genomics*.

[17] Nuss A. B., Mathew M. G., Gulia-Nuss M. (2016). Genomic insights into the Ixodes scapularis tick vector of Lyme disease. *Nature Communications*.

[18] de la Fuente J., Waterhouse R. M., Sonenshine D. E. (2016). Tick Genome Assembled: New Opportunities for Research on Tick-Host-Pathogen Interactions. *Frontiers in Cellular and Infection Microbiology*.

[19] Madden R. D., Sauer J. R., Dillwith J. W. (2002). A Proteomics Approach to Characterizing Tick Salivary Secretions. *Experimental and Applied Acarology*.

[20] Maya-Monteiro C. M., Daffre S., Logullo C. (2000). HeLp, a heme lipoprotein from the hemolymph of the cattle tick, Boophilus microplus. *The Journal of Biological Chemistry*.

[21] Díaz-Martín V., Manzano-Román R., Valero L., Oleaga A., Encinas-Grandes A., Pérez-Sánchez R. (2013). An insight into the proteome of the saliva of the argasid tick Ornithodoros moubata reveals important differences in saliva protein composition between the sexes. *Journal of Proteomics*.

[22] Oliveira C. J., Anatriello E., de Miranda-Santos I. K. (2013). Proteome of Rhipicephalus sanguineus tick saliva induced by the secretagogues pilocarpine and dopamine. *Ticks and Tick-Borne Diseases*.

[23] Valenzuela J. G., Francischetti I. M. B., Pham V. M., Garfield M. K., Mather T. N., Ribeiro J. M. C. (2002). Exploring the sialome of the tick Ixodes scapularis. *The Journal of Experimental Biology*.

[24] Sonenshine E. D. (1991). *Biology of Ticks*.

[25] Sonenshine D. E., Hynes W. L. (2008). Molecular characterization and related aspects of the innate immune response in ticks. *Frontiers in Bioscience*.

[26] Soberanes N. C., Santamaría M. V., Fragoso H. S., García Z. V. (2002). First case reported of amitraz resistance in the cattle tick Boophilus microplus in Mexico. *Journal of Visualized Experiments*.

[27] Stoepler T. M., Castillo J. C., Lill J. T., Eleftherianos I. (2012). A Simple Protocol for Extracting Hemocytes from Wild Caterpillars. *Journal of Visualized Experiments*.

[28] Stone B. F., Haydock K. P. (1962). A method for measuring the acaricide susceptibility, of the cattle tick Boophilus microplus (Can.). *Bulletin of Entomological Research*.

[29] Bradford M. M. (1976). Rapid and sensitive method for the quantitation of microgram quantities of protein utilizing the principle of protein-dye binding. *Analytical Biochemistry*.

[30] Miranda-Miranda E., Murillo-Sanchez M. H., Cossio-Bayugar R. (2008). Expression of a Haemonchus contortus cysteine protease in the baculovirus system. *Electronic Journal of Biotechnology*.

[31] Hood R. (2003). *Handbook of Detection of Enzymes on Electrophoretic Gels*.

[32] Sonenshine D. E., Macaluso K. R. (2017). Microbial invasion vs. tick immune regulation. *Frontiers in Cellular and Infection Microbiology*.

[33] Maya-Monteiro C. M., Daffre S., Logullo C. (2000). HeLp, a heme lipoprotein from the hemolymph of the cattle tick, Boophilus microplus. *The Journal of Biological Chemistry*.

[34] Sorkin A. (2001). Internalization of the epidermal growth factor receptor: Role in signalling. *Biochemical Society Transactions*.

[35] Enkhbayar P., Kamiya M., Osaki M., Matsumoto T., Matsushima N. (2004). Structural Principles of Leucine-Rich Repeat (LRR) Proteins. *Proteins: Structure, Function, and Genetics*.

[36] Kobe B., Kajava A. V. (2001). The leucine-rich repeat as a protein recognition motif. *Current Opinion in Structural Biology*.

[37] Gay N. J., Packman L. C., Weldon M. A., Barna J. C. J. (1991). A leucine-rich repeat peptide derived from the Drosophila Toll receptor forms extended filaments with a *β*-sheet structure. *FEBS Letters*.

[38] Rothberg J. M., Jacobs J. R., Goodman C. S., Artavanis-Tsakonas S. (1990). slit: An extracellular protein necessary for development of midline glia and commissural axon pathways contains both EGF and LRR domains. *Genes & Development*.

[39] Spadaro F., Cecchetti S., Fantuzzi L. (2017). Macrophages and phospholipases at the intersection between inflammation and the pathogenesis of hiv-1 infection. *International Journal of Molecular Sciences*.

[40] Greenburg A. G., Kim H. W. (2004). Hemoglobin-based oxygen carriers. *Critical Care (London, England)*.

[41] Umeda K., Heike T., Nakata-Hizume M. (2006). Sequential analysis of *α*- and *β*-globin gene expression during erythropoietic differentiation from primate embryonic stem cells. *Stem Cells*.

[42] Egawa T., Yeh S.-R. (2005). Structural and functional properties of hemoglobins from unicellular organisms as revealed by resonance Raman spectroscopy. *Journal of Inorganic Biochemistry*.

[43] Rubin H. (1996). Serine protease inhibitors (serpins): Where mechanism meets medicine. *Nature Medicine*.

[44] Tanaka A. S., Andreotti R., Gomes A., Torquato R. J. S., Sampaio M. U., Sampaio C. A. M. (1999). A double headed serine proteinase inhibitor - Human plasma kallikrein and elastase inhibitor - From Boophilus microplus larvae. *International Journal of Immunopharmacology*.

[45] Tirloni L., Reck J., Terra R. M. S. (2014). Proteomic analysis of cattle tick Rhipicephalus (Boophilus) microplus saliva: A comparison between partially and fully engorged females. *PLoS ONE*.

[46] Janadaree Bandara K. M. U., Parakrama Karunaratne S. H. P. (2017). Mechanisms of acaricide resistance in the cattle tick Rhipicephalus (Boophilus) microplus in Sri Lanka. *Pesticide Biochemistry and Physiology*.

[47] Miller T. A. (1988). Mechanisms of resistance to pyrethroid insecticides. *Parasitology Today*.

[48] Metcalf R. L. (1989). Insect resistance to insecticides. *Journal of Pesticide Science*.

[49] He H., Chen A. C., Davey R. B., Ivie G. W. (2002). Molecular cloning and nucleotide sequence of a new P450 gene, CYP319A1, from the cattle tick, Boophilus microplus. *Insect Biochemistry and Molecular Biology*.

[50] Cossío-Bayúgar R., Miranda-Miranda E., Ortìz-Nàjera A., Neri-Orantes S., Olvera-Valencia S. (2008). Cytochrome P-450 monooxygenase gene expression supports a multifactorial origin fro acaricide resistence in Rhipicephalus microplus. *Research Journal of Parasitology*.

[51] Aïzoun N., Aïkpon R., Padonou G. (2013). Mixed-function oxidases and esterases associated with permethrin, deltamethrin and bendiocarb resistance in Anopheles gambiae s.l. in the south-north transect Benin, West Africa. *Parasites & Vectors*.

[52] David J., Ismail H. M., Chandor-Proust A., Paine M. J. (2013). Role of cytochrome P450s in insecticide resistance: impact on the control of mosquito-borne diseases and use of insecticides on Earth. *Philosophical Transactions of the Royal Society B: Biological Sciences*.

[53] Donohue K. V., Khalil S. M., Mitchell R. D., Sonenshine D. E., Michael Roe R. (2008). Molecular characterization of the major hemelipoglycoprotein in ixodid ticks. *Insect Molecular Biology*.

[54] Tilman D. (2000). Causes, consequences and ethics of biodiversity. *Nature*.

[55] Stearns S. C. (1989). Trade-offs in life-history evolution. *Functional Ecology*.

[56] Roff D. A., Fairbairn D. J. (2007). The evolution of trade-offs: where are we?. *Journal of Evolutionary Biology*.

